# Pairwise Kinship Analysis by the Index of Chromosome Sharing Using High-Density Single Nucleotide Polymorphisms

**DOI:** 10.1371/journal.pone.0160287

**Published:** 2016-07-29

**Authors:** Chie Morimoto, Sho Manabe, Takahisa Kawaguchi, Chihiro Kawai, Shuntaro Fujimoto, Yuya Hamano, Ryo Yamada, Fumihiko Matsuda, Keiji Tamaki

**Affiliations:** 1 Department of Forensic Medicine, Kyoto University Graduate School of Medicine, Kyoto, Japan; 2 Unit of Human Disease Genomics, Center for Genomic Medicine, Kyoto University Graduate School of Medicine, Kyoto, Japan; 3 Forensic Science Laboratory, Kyoto Prefectural Police Headquarters, Kyoto, Japan; 4 Unit of Statistical Genetics, Center for Genomic Medicine, Kyoto University Graduate School of Medicine, Kyoto, Japan; Universitat Pompeu Fabra, SPAIN

## Abstract

We developed a new approach for pairwise kinship analysis in forensic genetics based on chromosomal sharing between two individuals. Here, we defined “index of chromosome sharing” (*ICS*) calculated using 174,254 single nucleotide polymorphism (SNP) loci typed by SNP microarray and genetic length of the shared segments from the genotypes of two individuals. To investigate the expected *ICS* distributions from first- to fifth-degree relatives and unrelated pairs, we used computationally generated genotypes to consider the effect of linkage disequilibrium and recombination. The distributions were used for probabilistic evaluation of the pairwise kinship analysis, such as likelihood ratio (LR) or posterior probability, without allele frequencies and haplotype frequencies. Using our method, all actual sample pairs from volunteers showed significantly high LR values (i.e., ≥ 10^8^); therefore, we can distinguish distant relationships (up to the fifth-degree) from unrelated pairs based on LR. Moreover, we can determine accurate degrees of kinship in up to third-degree relationships with a probability of > 80% using the criterion of posterior probability ≥ 0.90, even if the kinship of the pair is totally unpredictable. This approach greatly improves pairwise kinship analysis of distant relationships, specifically in cases involving identification of disaster victims or missing persons.

## Introduction

Investigation of DNA polymorphisms, such as short tandem repeats (STRs) or single nucleotide polymorphisms (SNPs), can be used to identify familial relationships. In forensic genetics, unknown human remains, such as those used for disaster victim identification (DVI) or missing person identification (MPI), can be identified by kinship analysis between the unknown DNA sample and a reference DNA sample. In modern society, people have fewer children and relatives. Since there are sometimes cases without reference profiles of first-degree relatives (i.e., parent-child and siblings), it is not uncommon to identify a person by using only a single reference sample from a more distant relative (i.e., grandchild, nephew, and first cousin).

In current forensic casework, DNA-typing systems of 15 STR loci are commonly used for kinship analysis. However, pairwise kinship analysis using these STR-typing systems is only feasible for the assessment of first-degree relatives [1]. SNPs have recently received attention for kinship determination in forensic fields [2–4]. We can currently analyze large numbers of SNP loci simultaneously by DNA microarray [5, 6] or next-generation sequencing [7, 8]. Analyzing genome-wide SNP data allows determination of how much heredity information is shared between two individuals and is useful for kinship determination, even for distant relationships. In previous studies, fifth-degree relative pairs could be readily distinguished from unrelated individuals by using genotype data from high-density SNPs [5, 6]. Kling et al [5] suggested that relationships as distant as second cousins (fifth-degree relationships) could be readily distinguished from both the unrelated and other relationships by calculating the posterior probabilities using genotype data from high-density SNP microarrays. Lareu et al [6] demonstrated the usefulness of genome-wide SNP genotyping to analyze claimed second cousins by counting the number of shared SNPs between two people. However, since considerable linkage disequilibrium (LD) between SNPs is ignored in these studies, these methods may be subject to false positives.

In this study, we developed a new approach for pairwise kinship analysis using 174,254 autosomal SNP loci in the HumanCore-12 BeadChip or HumanCore-24 BeadChip (Illumina, San Diego, CA, USA) and focused on chromosomal sharing between two individuals. In previous studies, rigorous identity by descent (IBD) segments on chromosomes were estimated using statistical methods (e.g., Hidden Markov Models) [9–12]. However, the purpose of these studies was IBD mapping and not kinship analysis in forensic genetics. Our approach uses “index of chromosome sharing” (*ICS*) between two individuals to determine the degree of kinship. The *ICS* value is simply calculated on the basis of identity by state (IBS) regions between two individuals and the genetic length [i.e., centi-Morgan (cM)] of these regions and results in probabilistic values [i.e., likelihood ratio (LR) or posterior probability]. Therefore, this approach is intuitive for non-experts of forensic genetics (e.g., police officers, judges, and juries).

To calculate LR or posterior probability, we proposed a method to compare the difference in the possibility of obtaining an *ICS* value in each relationship. Allele or haplotype frequencies are not used in this method; therefore, LD does not need to be considered for calculating LR or posterior probability. To increase calculation accuracy, the expected *ICS* distributions in each relationship need to be estimated. Therefore, we used computational genotypes of related and unrelated (UN) pairs for estimating the distributions. Related pairs included first- to fifth-degrees of collateral (C) relationships and first- to third-degrees of lineal (L) relationships. Computational genotypes were generated by using estimated SNP haplotypes and considering recombination between each SNP. These SNP haplotypes were estimated by the Shape-IT algorithm [13] using 1498 Japanese individuals to incorporate the effect of LD into computational genotypes.

We then validated the new method by investigating the accuracy of kinship determination. For the validation, we collected actual sample pairs from families of volunteers in each targeted relationship. In general, kinship analysis is performed to investigate whether an individual pair has a predicted relationship or is UN by calculating LR; therefore, we investigated whether relationships between up to fifth-degree relatives could be distinguished from UN pairs based on LR. To obtain more effective evidence, we also investigated the posterior probability from Bayes’ theorem, assuming all degrees of relationships. By using the posterior probability, we can determine the degree of kinship from an *ICS* value, even if there is no predicted relationship between the pair.

## Results

### Overview of a new approach for pairwise kinship analysis

**Fig 1:**
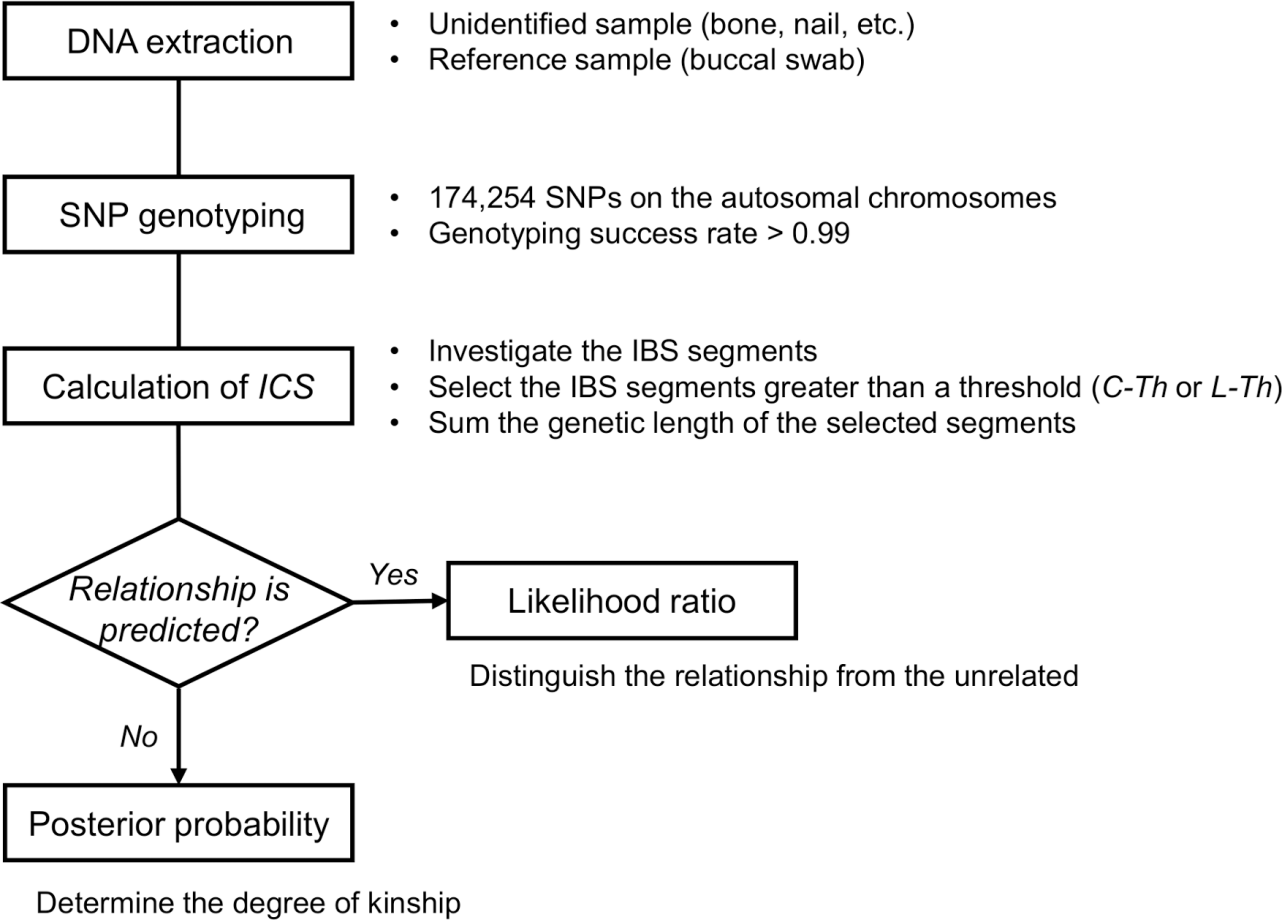
Overview of the method for pairwise kinship analysis proposed in this study. *ICS* is the index of chromosome sharing. *C-Th* and *L-Th* indicate the most appropriate thresholds for collateral relatives and for lineal relatives, respectively.

Fig 1 provides an overview of the method for pairwise kinship analysis proposed in this study. First, DNA is extracted from the samples of both the remains and the reference. Targeted relationships are first- to fifth-degrees of C relationships and first- to third-degrees of L relationships. Second, genotyping of 174,254 SNPs is performed by DNA microarray. Genotypes of samples delivering high-quality typing results (i.e., genotyping success rates > 0.99) are used for the following steps. Third, using the genotypes of two individuals from the 174,254 SNPs on the autosomal chromosomes, we calculate the *ICS* value. In the *ICS* calculation, we investigate the genetic length (cM) of all IBS segments between the two individuals, and then sum the genetic length of the IBS segments greater than a given threshold (*Th*). The most appropriate *Th* (i.e., *C-Th* for C relatives and *L-Th* for L relatives) has been determined by using computationally generated genotypes. Finally, we probabilistically evaluate the relationship of the pair by LR or posterior probability calculated by the probability density of the *ICS* distribution in each relationship. When the reference has the predicted relationship, LR is calculated to distinguish the relationship from the UN individuals. Alternatively, when there is no predicted relationship between the pair, posterior probability is calculated based on Bayes’ theorem to determine the degree of kinship.

### The *ICS* between individual pairs

*ICS* between two individuals was calculated by the summation of the genetic length of the IBS segments greater than *C-Th* or *L-Th*. We denoted *ICS* for C relatives as *ICS*_*C*_ and *ICS* for L relatives as *ICS*_*L*_. To determine *C-Th* and *L-Th*, the summation of genetic length greater than *Th* was defined as the function of *Th*: *ics*(*Th*). We calculated the *ics*(*Th*) values from computationally generated genotypes from 249 families synthesized from the genotype data of a community-based prospective multi-omics cohort (the Nagahama Study), changing *Th* from 0 to 63 cM (S1 Fig). These families included five types of C relative pairs, three types of L relative pairs, and UN individuals. The five types of C relationships were sibling (C-1), uncle-nephew (C-2), first cousin (C-3), first cousin once removed (C-4), and second cousin (C-5). The three types of L relationships were parent-child (L-1), grandparent-grandchild (L-2), and great-grandparent-great-grandchild (L-3). Although relationships with the same degree of kinship are expected to have almost the same *ICS* values, each relationship may actually have a different *ICS* distribution, because the chromosomal inheritance pattern is different, even in cases with the same degree of kinship (i.e., C-2 and L-2). Therefore, we treated C and L groups separately.

**Fig 2:**
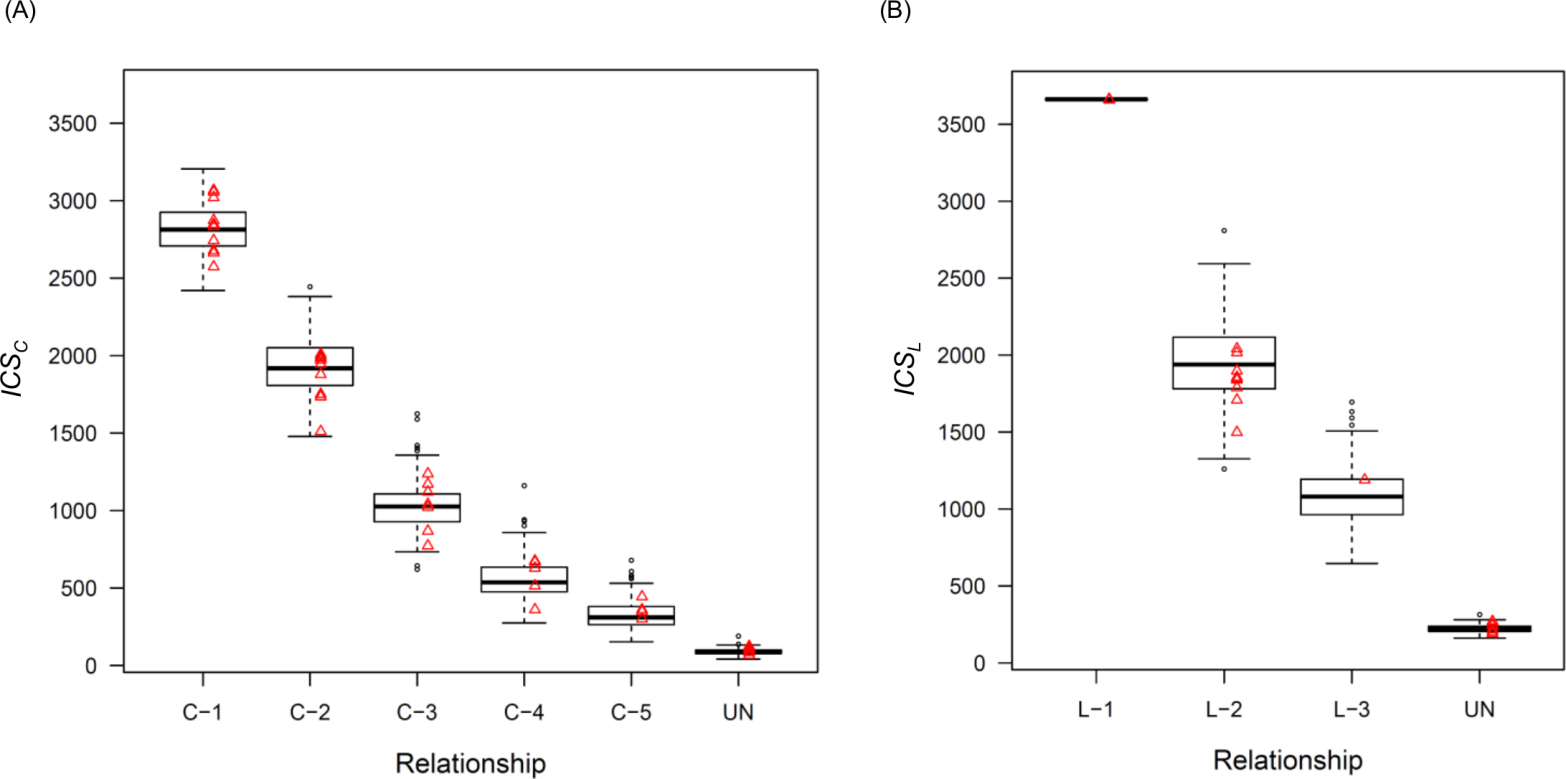
The expected *ICS* variability for each relationship. Boxplots indicate the variability from the computationally generated genotypes. Triangular plots indicate the values from half of the actual samples. (A) *ICS*_*C*_ variability for collateral relatives using only IBS segments longer than 4 cM and (B) *ICS*_*L*_ variability for lineal relatives using only IBS segments longer than 3 cM.

By using the area under the curve (AUC) for the receiver operating characteristic (ROC) curve, we adopted 4 cM and 3 cM as *C-Th* and *L-Th*, respectively (S1 Table). There were large differences in both *ics*(4) (i.e., *ICS*_*C*_) and *ics*(3) (i.e., *ICS*_*L*_) between each relationship, and these *ICS*_*C*_ and *ICS*_*L*_ values became lower as the pair exhibited a more distant relationship between both C relatives (Fig 2A) and L relatives (Fig 2B).

To evaluate the accuracy of the estimated *ICS* (*ICS*_*C*_ or *ICS*_*L*_) distribution from computationally generated pairs, we also investigated *ICS* values of actual individual pairs in each relationship, which were genotyped by the HumanCore-12 BeadChip or HumanCore-24 BeadChip. Genotyping success rates of all samples were > 0.99, which was recommended as good quality by the manufacturer. Using the *ICS* values from half of the sample genotypes, we confirmed that there were no outliers by comparing these values (triangular plots in Fig 2) to those of the simulated pairs (boxplots in Fig 2).

For L-1, all loci had at least one shared allele, and the *ICS*_*L*_ values of all pairs were the same as 3662.522, which was the maximum *ICS* value when all SNPs were shared between a pair in simulated genotypes, because simulated genotypes did not consider uncalled SNPs and typing errors. Despite *ICS*_*L*_ values of actual sample pairs being slightly lower than the maximum value due to typing errors in the DNA microarray, there was little difference between *ICS*_*L*_ values from actual sample pairs and those from simulated pairs. Because the *ICS*_*L*_ values in L-1 were completely separated from other relationships, even those with the same degree of kinship (C-1), we could distinguish L-1 from other relationships easily. Therefore, we removed L-1 from further analyses of L relatives.

### Estimating the *ICS* distribution

**Fig 3:**
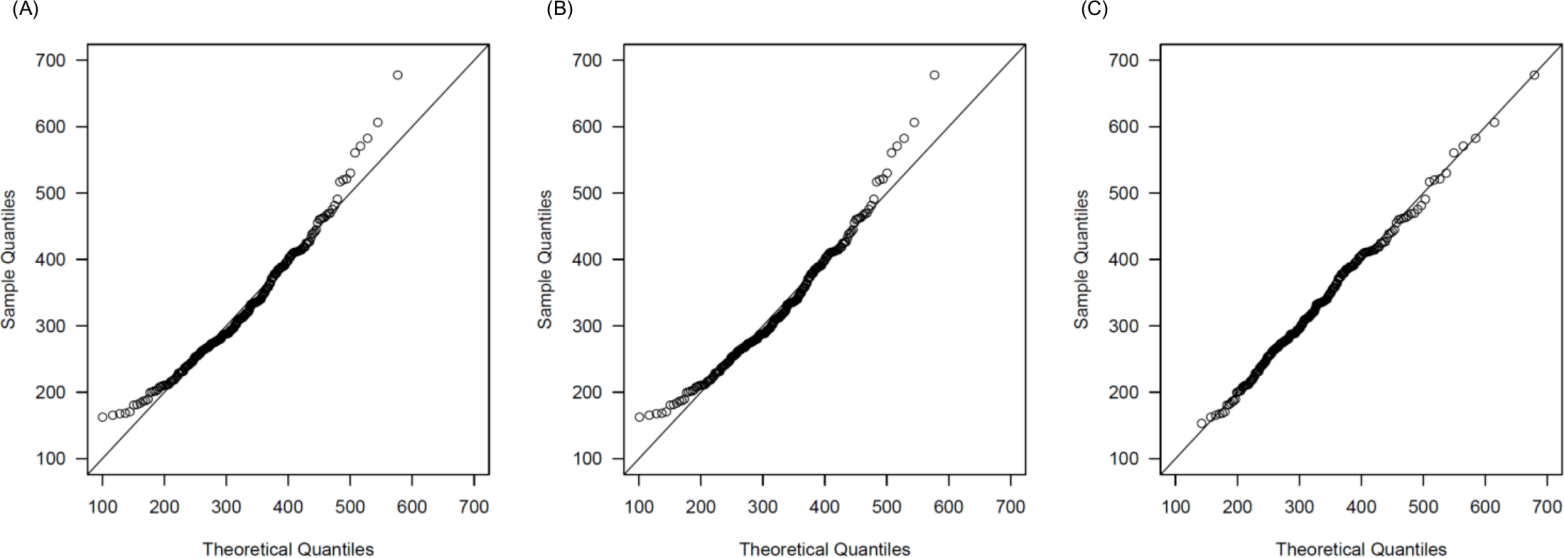
Q-Q plots for the *ICS*_*C*_ values calculated by the genotypes of computationally generated C-5. These plots are assumed as (A) the normal distribution, (B) the truncated-normal distribution, and (C) the log-normal distribution.

For kinship determination using *ICS*, we needed to know the expected distribution of *ICS* values in each relationship. Using the *ICS* values of simulated pairs, we estimated the *ICS* distribution in each relationship, except for L-1. We proposed three models (i.e., normal distribution, truncated-normal distribution, and log-normal distribution) for distribution of the *ICS* values due to the shape of *ICS* values for simulated pairs. For example, Fig 3 shows the quantile-quantile (Q-Q) plots of the three models in C-5. The Q-Q plots indicated that the log-normal distribution was the best-fitted model, while the normal and the truncated-normal distributions differed from the observed *ICS*_*C*_ values. In other relationship pairs (i.e., C-1 to C-4; L-2, and L-3), log-normal distributions were also fitted.

We also calculated the Akaike Information Criterion (AIC) values for each model in order to evaluate which model was the most appropriate. The model with the lowest AIC was considered the best-fit model. In C relatives, the AIC values from the normal distribution model, truncated-normal distribution model, and log-normal distribution model were 17992.85, 17992.78, and 17946.81, respectively. In L relatives, the AIC values from the three models were 9073.437, 9073.437, and 9068.232, respectively. The log-normal distribution model indicated the lowest AIC in both C relatives and L relatives. Therefore, we modeled the *ICS* distribution obtained from the simulated genotypes of C relatives and L relatives as a log-normal distribution. The estimated parameters (μ and σ) used in log-normal distributions for each relationship are shown in S2 Table.

In order to evaluate whether the modeled distribution of *ICS* values was appropriate, we compared the *ICS* values obtained from the SNP genotypes from half of the actual sample pairs (triangular plots in Fig 2) with the estimated distribution in each relationship by z test. All *p*-values were > 0.05; therefore, there was no significant difference between the estimated *ICS* distribution and the *ICS* values of the actual sample pairs. Thus, we considered that the estimated distribution properly reflected the actual *ICS* variability.

### LR by comparing the *ICS* distributions of related and UN pairs

In general, kinship analysis is performed to investigate whether an individual pair has a predicted relationship or is UN by calculating LR. Using the estimated *ICS* distribution (i.e., log-normal distribution), we investigated the probability density obtained from an *ICS* value by hypothesizing a specific relationship between an individual pair. The difference in the probability density between two hypothesized relationships could be expressed as a LR. Using the rest of the actual sample pairs, we calculated the LR values based on the estimated *ICS* distribution. The minimum, median, and maximum LR values are shown in Table 1. Significantly high LR values were obtained from all related pairs, even C-5. Therefore, this suggested that it was possible to distinguish up to fifth-degree relationships for C relatives and third-degree relationships for L relatives from UN pairs using the method proposed in this study.

**Table 1:** Three quantiles of LR values for the actual sample pairs obtained by comparing the true relationship with the UN.

**Collateral relatives**				
**Relationship**	**N**^**a**^	**LR**		
		**Minimum**	**Median**	**Maximum**
C-1	10	1.76 × 10^52^	6.62 × 10^55^	7.16 × 10^57^
C-2	8	2.66 × 10^39^	2.87 × 10^43^	1.38 × 10^47^
C-3	7	1.37 × 10^24^	1.93 × 10^27^	6.82 × 10^33^
C-4	5	2.76 × 10^9^	2.28 × 10^16^	1.05 × 10^18^
C-5	3	1.63 × 10^8^	2.74 × 10^10^	6.86 × 10^18^
**Lineal relatives**				
**Relationship**	**N**^**a**^	**LR**		
		**Minimum**	**Median**	**Maximum**
L-2	8	6.52 × 10^69^	7.23 × 10^88^	2.60 × 10^94^
L-3	1	1.16 × 10^45^		

^a^ N indicates the number of pairs.

In order to investigate whether the UN pairs could be distinguished from distant relationships, we calculated the LR values assuming *H*_1_ was that the pair was C-5 and *H*_2_ was that the pair was UN using the *ICS*_*C*_ values of UN. The three quantiles were 5.53 × 10^−7^, 4.50 × 10^−5^, and 1.10 × 10^−3^. We also calculated LR values in L relatives (i.e., L-3) using the *ICS*_*L*_ values of UN. The three quantiles were 1.25 × 10^−24^, 8.16 × 10^−20^, and 8.19 × 10^−16^. These values suggested that we were able to discriminate UN from both C-5 and L-3.

### Calculation of the posterior probabilities assuming all degrees of relationship

To determine the degree of kinship from an *ICS* value, we calculated the posterior probability from Bayes’ theorem assuming all degrees of relationship. The flat values of the prior probabilities were used, because we assumed that all hypotheses were equally likely before obtaining the genotype data. Although there are a number of publications concerning the strength of evidence, judgment criteria have not been standardized. In this study, we adopted Hummel’s predicates [14] as the criteria of judgment for kinship determination. The predicates are as follows:

1. a relationship is “practically proven” if the posterior probability ≥ 0.998
2. a relationship is “highly likely” if the posterior probability is ≥ 0.99 and < 0.998
3. a relationship is “very likely” if the posterior probability is ≥ 0.95 and < 0.99
4. a relationship is “likely” if the posterior probability is ≥ 0.90 and < 0.95
5. a relationship is “undetermined” if the posterior probability is < 0.90

**Fig 4:**
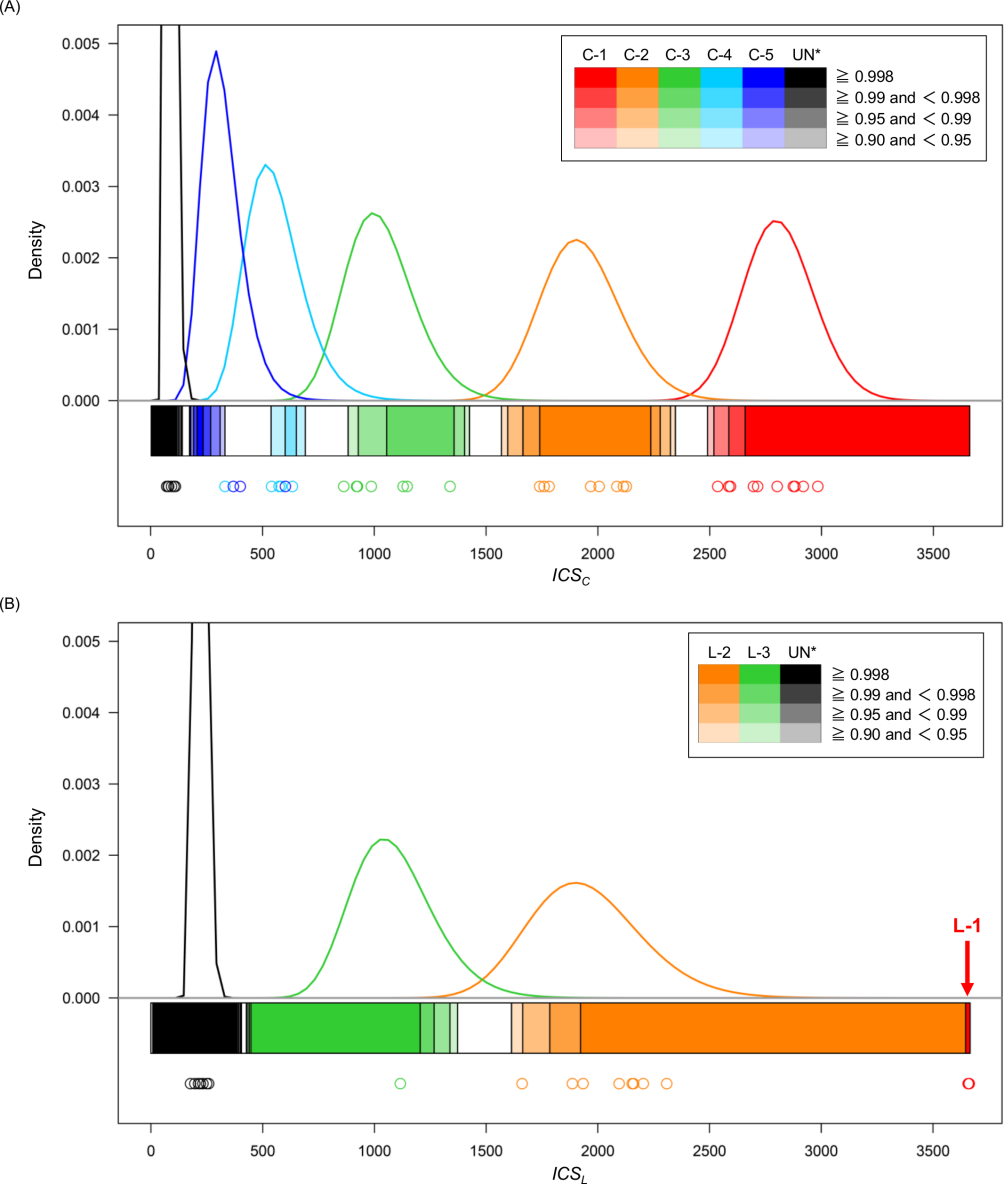
The range of *ICS* values in each Hummel’s predicate for kinship determination. *ICS* values for (A) collateral relatives and (B) lineal relatives. Solid lines indicate the estimated log-normal distributions of the *ICS*_*C*_ and *ICS*_*L*_ values. The colors in the bar under the distributions indicate the range of the *ICS*_*C*_ and *ICS*_*L*_ values that can be obtained from the posterior probabilities corresponding to each Hummel’s predicate for each relationship. The plots indicate the *ICS*_*C*_ and *ICS*_*L*_ values calculated by the rest of the actual sample pairs. All actual L-1 pairs were almost equal to the maximum value of *ICS*_*L*_ (i.e., 3662.522) and are indicated in red. * The distribution of UN is partially displayed because the density of the estimated distribution of UN is high.

We first investigated the *ICS* range in each Hummel’s predicate using the estimated distribution for C relatives (Fig 4A) and L relatives (Fig 4B). The range is indicated by the bar under the distributions in each relationship and separated by specific colors. The deeper the color of the relationship, the higher the posterior probabilities. Table 2 shows the percentage of the distributions having posterior probabilities ≥ 0.998, ≥ 0.99, ≥ 0.95, and ≥ 0.90. For the C relatives, > 80% of C-1 and C-2 demonstrated that the posterior probabilities were ≥ 0.998. For C-3 and more distant relationships, it was difficult to obtain posterior probabilities ≥ 0.998, but > 80% of C-3 demonstrated posterior probabilities ≥ 0.90. However, it was difficult to distinguish C-4 from close relationships, because the percentages of these relationships demonstrating the high posterior probabilities (≥ 0.90) were 37.4%. Similarly, it was also difficult to distinguish C-5 from close relationships, because the percentages of these relationships demonstrating high posterior probabilities (≥ 0.90) were 56.9%. The percentage associated with C-5 was actually lower due to the lack of more distant relationships (e.g., sixth-degree relatives). For the L relatives, more than half of L-2 and L-3 demonstrated posterior probabilities of ≥ 0.998, and > 90% of pairs demonstrated posterior probabilities of ≥ 0.90. The identification rate of L-3 was actually lower due to the lack of more distant relationships (e.g., fourth-degree relatives). It may be difficult to determine more distant relationships not being considered in this study (e.g., sixth-degree C relationships or fourth-degree L relationships), but there are few opportunities to identify such relationships in forensic casework. In both C and L relatives, almost all UN pairs demonstrated posterior probabilities of ≥ 0.998; therefore, UN pairs were rarely predicted as certain relatives using our method.

**Table 2:** Percentages of the posterior probabilities ≥ 0.998, 0.99, 0.95, and 0.90 for the distributions of each relationship.

**Collateral relatives**				
**Relationship**	Posterior probability*			
	**≥ 0.998**	**≥ 0.99**	**≥ 0.95**	**≥ 0.90**
C-1	83.0%	92.6%	97.2%	98.3%
C-2	80.6%	90.5%	95.7%	97.0%
C-3	0.0%	38.0%	71.6%	81.6%
C-4	0.0%	0.0%	11.5%	37.4%
C-5	6.66%	24.1%	46.4%	56.9%
UN	89.2%	94.8%	97.6%	98.4%
**Lineal relatives**				
**Relationship**	Posterior probability*			
	**≥ 0.998**	**≥ 0.99**	**≥ 0.95**	**≥ 0.90**
L-2	51.6%	73.2%	87.7%	91.9%
L-3	75.4%	83.7%	90.4%	92.7%
UN	99.99997%	> 99.99999%	≈ 100%	≈ 100%

UN, unrelated.

* The flat values of the prior probabilities were used.

We also calculated the posterior probabilities using half of the actual sample pairs (the same pairs that are shown in Table 1). The *ICS* values of the pairs were plotted in Fig 4, and probability densities, which were used for calculating the posterior probabilities, were obtained from the Y-axis of the graph in Fig 4. Table 3 shows the number of samples categorized by the range of posterior probabilities based on Hummel’s predicates. Detailed posterior probabilities are shown in S3 Table. For C relatives, almost all pairs of C-1, C-2, C-3, C-4, and UN supported true relationships (i.e., posterior probabilities were ≥ 0.90). However, three C-5 pairs demonstrated that the posterior probability of the true relationship was < 0.90 (i.e., categorized as “undetermined”). One of these three pairs demonstrated that the posterior probability of C-4 was 0.951 (i.e., falsely predicted as “very likely” C-4). For L relatives, all pairs of L-2, L-3, and UN demonstrated posterior probabilities of ≥ 0.90 (i.e., predicted as “likely” or higher); therefore, each relationship was determined correctly. These results suggested that this method has high discriminatory ability for comparing the closeness of up to third-degree relationships.

**Table 3:** The number of samples categorized by the range of posterior probabilities based on Hummel’s predicates.

**Collateral relatives**						
**Relationship**	**N**^**a**^	Posterior probability*				
		**≥ 0.998**	**≥ 0.99**	**≥ 0.95**	**≥ 0.90**	< **0.90**
C-1	10	7	2	1		
C-2	8	8				
C-3	7		3	1	2	1
C-4	5			1	3	1
C-5	3					3
UN	7	7				
**Lineal relatives**						
**Relationship**	**N**^**a**^	Posterior probability*				
		**≥ 0.998**	**≥ 0.99**	**≥ 0.95**	**≥ 0.90**	< **0.90**
L-2	8	6	1		1	
L-3	1	1				
UN	7	7				

UN, unrelated.

* The flat values of the prior probabilities were used.

^a^ N indicates the number of pairs.

## Discussion

We developed a new approach for pairwise kinship analysis by using the *ICS* value, which was calculated on the basis of IBS regions between two individuals. Although *ICS* value is not the total genetic length of actual chromosomal sharing (i.e., IBD segments), it demonstrates different distributions in each relationship. We can discriminate distant relationships up to the fifth-degree from UN individuals. Moreover, we can determine accurate degrees of kinship up to the third-degree in both C and L groups, even if the kinship of the pair is totally unpredictable. We also confirmed that the *ICS* values calculated from actual samples had the same *ICS* distributions obtained from computationally generated individuals, which was a different result from that reported in a previous study based on theoretical simulation [15]. Therefore, we have developed a realistic method for pairwise kinship analysis based on chromosomal sharing.

Here, we proposed a method for calculating LR and posterior probability by comparing the probability density of an *ICS* value in each relationship, but not using the allele or haplotype frequencies. Sun et al [16] proposed a method of calculating posterior probability for the identification of distant relative pairs by using allele or haplotype frequencies and considering LD. In the near future, we will evaluate which method of probabilistic calculation is the most accurate for kinship analysis.

DNA microarrays used for this approach (i.e., HumanCore-12 BeadChip or HumanCore-24 BeadChip) can analyze 48 samples simultaneously at a low cost (≈$80 USD per sample). Moreover, all processes from DNA extraction to data analysis take only 3 days. Thus, this typing method is both cost- and time-effective for forensic casework, especially in cases such as DVI, where large numbers of samples need to be analyzed. Currently, pairwise kinship analysis using DNA-typing systems of 15 STR loci is only feasible for the assessment of first-degree relatives. While we can discriminate 69.8% of first-degree relatives (i.e., siblings) from UN, only 4.07% of second-degree relatives (i.e., half siblings) can be discriminated [17]. However, unknown human remains sometimes cannot be identified due to there being no reference profile for close relatives, such as first-degree relatives. In 2011, a tsunami following the Great East Japan Earthquake took the lives of approximately 16,000 people. Although identification of victims was performed using various methods (e.g., from information about clothing, fingerprints, dental records, and DNA analysis), > 70 unidentified victims are still awaiting return to their families [18]. The new approach proposed in this study has the potential to properly identify these victims.

However, there are areas of our approach that need to be improved upon prior to application in practical forensic cases. First, additional studies are required to assess the versatility of microarray typing. In this study, we used 200 ng of DNA extracted from buccal swabs of the volunteers for the microarray; however, we often have access to smaller amounts of DNA and can obtain only degraded samples according to circumstances. We preliminarily confirmed that genotyping results with good quality (i.e., genotyping success rates > 0.99) were obtained using as little as 10 ng of DNA extracted from buccal swabs. We need to evaluate whether microarray typing can be applied to degraded forensic samples (e.g., nails, bones, or teeth).

Second, we currently have to choose from 174,254 SNPs in HumanCore-12 BeadChip or HumanCore-24 BeadChip (Illumina) when we apply this approach to actual casework. Any SNP panel can be used as long as a large-scale SNP genotype dataset is available for simulation. We expect that the length of IBS regions in autosomal chromosomes is less affected by increases or decreases in the number of SNPs. To enhance the versatility of the method, we will evaluate the effect of the set and number of SNPs on this approach.

This method is limited for use in Japanese populations, because other populations are expected to have different *ICS* distributions. If our approach is applied to a non-Japanese population, a large-scale SNP genotype dataset (i.e., >1000 individuals) for the population will be needed to generate distributions of *ICS* values. However, even if such a dataset for the population is not available, our approach may be worth evaluating, because genotype imputation can be performed in a comparatively cross-ethnic way [19]. Additionally, use of different datasets for simulations may result in variations in the estimated *ICS* distribution and the most appropriate *Th* value (i.e., *C-Th* and *L-Th*). Further consideration will be needed to confirm the quality of *C-Th* and *L-Th*.

Our approach is only useful for kinship analysis between two individuals and for applications to assess the minimal requirements depending on the number of DVI candidates. Additionally, we may need a new index other than *ICS* in cases where the references of two or more people are present. Furthermore, when there are many samples from both human remains and references, especially in DVI cases, it will be desirable to assess the results by comparing the remains of each individual with the entire reference, similar to the permanent method [20].

In conclusion, our approach using high-density SNP genotyping improved the accuracy for pairwise kinship analysis by using *ICS* values and performing probabilistic evaluation. Therefore, this approach can be applied to actual forensic casework involving personal identification for DVI and MPI cases.

## Methods

### High-density SNP genotyping

Buccal samples were collected from 67 Japanese individuals in five families by using a Buccal DNA Collector (Bode Technology, Lorton, VA, USA). These families contained nine types of relationships, including five types of C relationships, three types of L relationships, and UN individuals. Five types of C relationships included 20 sibling pairs (C-1), 17 uncle-nephew pairs (C-2), 14 first cousin pairs (C-3), 10 first cousin once removed pairs (C-4), and seven second cousin pairs (C-5). Three types of L relationships included 21 parent-child pairs (L-1), 17 grandparent-grandchild pairs (L-2), and two great-grandparent-great-grandchild pairs (L-3). We also collected samples from 15 UN pairs. These pairs were used regardless of sex, because we targeted only autosomal SNPs. All participants gave written informed consent, and this study was approved by the ethics committee of the Graduate School of Medicine of Kyoto University. Buccal-cell DNA was extracted using a QIAamp DNA Investigator kit (Qiagen, Venlo, Netherlands), and the extracted DNA was quantified using Quanti-iT PicoGreen dsDNA Assay kit (Invitrogen, Carlsbad, CA, USA). Each DNA sample normalized to a final DNA concentration of 50 ng/μL.

We genotyped all samples using the HumanCore-12 BeadChip or HumanCore-24 BeadChip according to manufacturer protocol. The genotyping platform adopted in this study was the Illumina Infinium HD Assay. SNP chips were scanned using iScan (Illumina) and analyzed using GenomeStudio (Illumina). Genotyping success rates of all samples were > 0.99, which is recommended by the manufacturer to ensure good quality. Half of the actual pairs were used to evaluate the estimated *ICS* distribution from simulated pairs, and the rest were used to validate the method proposed in this study.

HumanCore-12 BeadChip combines 12,602 InDels and 286,328 SNPs, of which 276,041 SNPs are from autosomal chromosomes and the remaining 10,287 SNPs include X and Y SNPs, mitochondrial SNPs, and non-informative SNPs (i.e., containing no information about their chromosomes from the manufacturer references). HumanCore-24 BeadChip is an update of the HumanCore-12 BeadChip and combines 12,663 InDels and 294,007 SNPs, of which 283,518 SNPs are from autosomal chromosomes and the remaining 10,489 SNPs include X and Y SNPs, mitochondrial SNPs, and non-informative SNPs. Results of InDels, X and Y SNPs, mitochondrial SNPs, and non-informative SNPs were not used in this study. We also removed autosomal SNPs without information concerning genetic map position in SNAP version 2.2 [21].

### Generating computer-based familial genotypes of high-density SNPs

**Fig 5:**
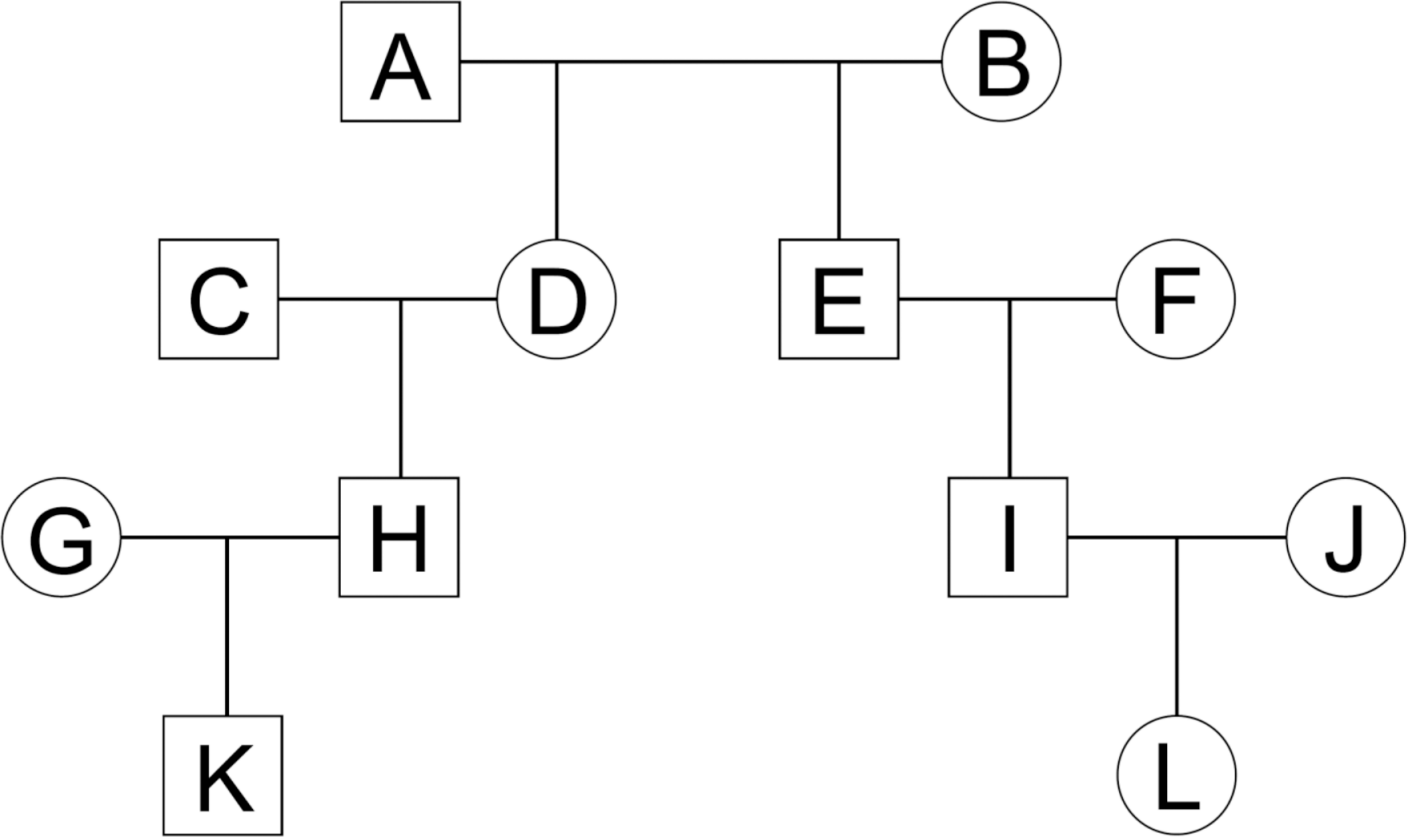
Pedigree consisted of twelve people.

We computationally synthesized a number of the familial genotypes shown in Fig 5. The family included five C relatives, three L relatives, and UN pairs. C relatives were C-1 (D-E), C-2 (D-I), C-3 (H-I), C-4 (H-L), and C-5 (K-L). L relatives were L-1 (A-E), L-2 (A-I), and L-3 (A-L). To synthesize realistic genotypes of the family, we generated founder haplotypes (i.e., A, B, C, F, G, and J in Fig 5) by considering the effect of LD and recombination. To generate these haplotypes, we used genotype data from the Nagahama Prospective Genome Cohort for Comprehensive Human Bioscience (the Nagahama Study), a community-based prospective multi-omics cohort study conducted by the Center for Genomic Medicine at Kyoto University. We used SNP data from 1616 individuals in the Nagahama Study, which were genotyped using the HumanOmni 2.5M-4 Beadchip (Illumina). Quality checks for this SNP data were performed using a series of quality control (QC) filters in a function of PLINK [22], which included individual genotyping success rate (> 0.95), SNP genotyping success rate (> 0.99), Hardy-Weinberg equilibrium (*p* > 1 × 10^−6^), and minor allele frequency (MAF) cutoffs (MAF > 0.01). Additionally, we did not use individually estimated first-degree relatives (i.e., parent-child and siblings) from PI-HAT to remove unexpected relationships from the genotype dataset.

After SNP data from 1498 individuals satisfied the QC, we estimated the haplotypes of these individuals for generating founder haplotypes in Fig 5. We used 174,254 autosomal SNPs as haplotype components. We did not select SNPs by MAF and genetic distances and used as many SNPs as possible to obtain rigorous IBS regions for all autosomal chromosomes for calculating *ICS*. These SNPs were included in both the HumanCore-12 BeadChip and the HumanCore-24 BeadChip, and genetic map positions (cM) of these SNPs were obtained from SNAP version 2.2 [21]. Haplotypes of the 1498 individuals with the 174,254 SNPs were estimated using the Shape-IT algorithm [13].

Since there were six founders in one family, we synthesized 249 familial genotypes using the estimated haplotypes of 1494 individuals as founder haplotypes that were randomly selected from 1498 individuals. We then generated the haplotypes of descendants by taking into account recombination events. We produced recombined chromosomes of founders by using the recombination rates between adjacent SNPs. Because the genetic map distances between SNPs targeted in this study were small, the distances and recombination rates were approximately equivalent. Therefore, we regarded the recombination rates as 1/100 of cM values (e.g., if the genetic distance between two SNPs was 1 cM, the recombination rate was 0.01). We then chose one recombined chromosome at random and assigned the chromosome to the descendant. Similarly, the haplotypes of all members in Fig 5 were generated with occurrence of recombination events by generations. All programs used for simulations and statistical analyses described below were performed using the statistical software R version 3.1.2 [23] and are provided through GitHub (https://github.com/ChieMorimoto/Kinship-analysis-by-ICS).

### Defining *ICS* between individual pairs

The *ICS* is a value based on IBS regions and reflects the total genetic length of chromosome sharing between two individuals. To calculate the *ICS* value, we first investigate the genetic length of all IBS regions, and then sum the genetic length of the IBS regions selected by a threshold for removing the effect of coincidental matches according to the following two subsections.

#### Investigating genetic length of the shared segments based on identity by state

We first order all 174,254 SNPs according to their chromosomal positions and then count the IBS states in each locus between two individuals. The IBS state has three possible outcomes: 0, 1, or 2. Uncalled SNPs in either individual are ignored in the following analysis. We choose “the shared segments” (i.e., the regions where 1 or 2 are continuously aligned). The genetic length of shared segments is expressed as the genetic distance, which is the difference between the genetic map position (cM) [21] of the first and last SNP of each shared segment. For example, assuming that the IBS state between a pair at ten SNPs is 2212010221, the shared segments are from the first SNP to the fourth SNP (i.e., 2212) and from the eighth SNP to the tenth SNP (i.e., 221). We do not regard isolated sharing, such as the sixth SNP, as a shared segment. The genetic length of “2212” represents the difference between the genetic map position of the first SNP and the fourth SNP. The genetic length of “221” similarly represents the difference between the genetic map position of the eighth SNP and the tenth SNP. We investigate the genetic length of all shared segments in all autosomal chromosomes.

#### Summing the genetic length of the shared segments selected by a threshold

Some of the shared segments obtained from the IBS state can be coincidental matches, because even UN pairs can share alleles by chance. For calculating *ICS* values, we have to exclude comparatively shorter shared segments in order to minimize the effect of coincidental matches. In contrast, if we exclude too many shared segments, we could miss the actual share of chromosomes.

We set a *Th* for the genetic length of the shared segments between two individuals and sum only the genetic length greater than the *Th*. The most appropriate *Th* value has been determined using computational genotypes of 174,254 SNPs. The summation under the most appropriate *Th* value is called the *ICS*, which is expected to become larger as an individual pair has a higher degree of kinship.

We used computationally synthesized genotypes of 174,254 SNPs to find the most appropriate *Th* value for discriminating each relationship. The summation of genetic length greater than the *Th* is defined as the function of *Th*: *ics*(*Th*). We calculated the *ics*(*Th*) values in each simulated pair, changing *Th* from 0 to 63 cM, which was the genetic length of the shortest autosomal chromosome (i.e., chromosome 21). We then assessed the performance of discrimination between close-relative pairs by using the AUC. Although relationships with the same degree of kinship are expected to have almost the same *ICS* values, each relationship may actually have a different *ICS* distribution, because the chromosomal inheritance pattern is different, even in cases with the same degree of kinship (i.e., C-2 and L-2). Therefore, we treated C and L groups separately in this study, including for ROC analysis. The five AUC values were calculated by five comparisons of collateral relatives (i.e., C-1 vs. C-2, C-2 vs. C-3, C-3 vs. C-4, C-4 vs. C-5, and C-5 vs. UN) per *Th*. We adopted the *Th* value that provided the maximum of the mean of five AUC values for discrimination of C relatives. Similarly, the three AUC values were calculated by three comparisons of L relatives (i.e., L-1 vs. L-2, L-2 vs. L-3, and L-3 vs. UN) per *Th*. We adopted the *Th* value that provided the maximum of the mean of three AUC values for discrimination of L relatives. These adopted *Th* values were the most appropriate for C relatives (i.e., *C-Th*) and for L relatives (i.e., *L-Th*). We termed the *ICS* value under *C-Th* as *ICS*_*C*_ and the *ICS* value under *L-Th* as *ICS*_*L*_.

### Estimating the *ICS* distribution

For kinship determination, we need to know the expected variability of *ICS* (*ICS*_*C*_ or *ICS*_*L*_) in each relationship. Therefore, we estimated the *ICS* probability distribution. We proposed three models of *ICS* distribution: normal distribution, truncated-normal distribution, and log-normal distribution. Truncated-normal distribution is the probability distribution of a normally distributed random variable whose value is bounded either below or above (or both). In this study, the random variable was *ICS*, and the limited range was from 0 to 3662.522, which is the maximum value of *ICS* when all SNPs are shared between a pair. The maximum value is approximately the sum of the genetic map distances (cM) in all autosomal chromosomes. These three models in one relationship need a location parameter (μ) and a scale parameter (σ). We estimated these parameters for nine types of relationships by maximum likelihood estimation using the *ICS* obtained from 249 simulated familial genotypes. We then compared the fit of these models using a Q-Q plot and calculating the AIC:

$$AIC=2k-2({log}_{e}ML)$$

where k is the number of parameters and *ML* is the maximum likelihood in each model. The AIC is used to assess the relative goodness of fit of different models for the same dataset. We separated the C and L relatives for AIC calculation, and UN pairs were included in both C and L groups. The model with the lowest AIC was considered the best-fit model, which was adapted as the *ICS* distribution.

To verify that the estimated *ICS* distribution was fit to those of the actual sample pairs, a z test was performed to compare the estimated distribution and *ICS* values calculated using half of the actual sample pairs in each relationship. A *p* < 0.05 was considered statistically significant.

### Calculating LR by comparing the *ICS* distributions between related and UN pairs

In general, kinship analysis is performed to investigate whether an individual pair has a predicted relationship or is UN by calculating a LR. Using a probability density of estimated *ICS* distributions, we can investigate the possibility of obtaining an *ICS* value by hypothesizing a specific relationship between an individual pair. The difference in probability density values between two hypothesized relationships can be expressed as a LR. The LR is the ratio of the conditional probability of obtaining an *ICS* value of a pair under a certain relationship (*H*_1_) and the conditional probability of obtaining an *ICS* value of the pair under another relationship (*H*_2_). Here, the LR is defined as follows:

$$LR=\frac{f(ICS|H_{1})}{f(ICS|H_{2})}$$ (1)

In this study, we calculated the LR values using *ICS* values obtained from the other half of the actual sample pairs in order to investigate our ability to discriminate a specific relationship from UN pairs. We hypothesized that *H*_1_ represents a true relationship and *H*_2_ represents UN individuals. For example, in a case of sibship analysis using an actual sibling pair, we first calculated the *ICS* value between the pair. We then obtained the probability density of the *ICS* value in the estimated distribution of a sibling (C-1) and the probability density of the *ICS* value in the estimated distribution of an UN individual. Finally, we calculated the ratio of the two densities as the LR value.

### Calculation of the posterior probabilities assuming all degrees of kinship

To determine the degree of kinship from an *ICS* value in a case where there was no predicted relationship between the pair, we calculated the posterior probability from Bayes’ theorem, assuming all degrees of relationship in both C and L relatives. The posterior probability of a certain relationship (*H*_*i*_) is defined as follows:

$$\mathrm{Pr}\left(H_{i}|ICS\right)=\frac{f\left(ICS|H_{i}\right)\mathrm{Pr}\left(H_{i}\right)}{\sum_{j=1}^{n}f\left(ICS|H_{j}\right)\mathrm{Pr}\left(H_{j}\right)}$$ (2)

where *n* denotes the number of assumed relationships and *H*_*j*_ (*j* = 1, 2, … *i*, … *n*) denotes the hypothesis that an individual pair is the *j*th relationship. *f*(*ICS*|*H*_*j*_) is the probability density of an *ICS* value obtained from the probability distribution in *H*_*j*_, and Pr(*H*_*j*_) is the prior probability under *H*_*j*_. The flat values of the prior probabilities were used because we assumed that all hypotheses were equally likely before obtaining the genotype data. Therefore, Eq 2 could be changed as follows:

$$\mathrm{Pr}\left(H_{i}|ICS\right)=\frac{f\left(ICS|H_{i}\right)}{\sum_{j=1}^{n}f\left(ICS|H_{j}\right)}$$ (3)

The posterior probabilities (i.e., Pr(*H*_*i*_|*ICS*)) were calculated in each assumed relationship of both C and L relatives.

Using actual sample pairs, which were also used for the LR calculation, we calculated the posterior probabilities of C and L relatives separately. In other words, when we targeted C relatives, the competing relationships were C-1, C-2, C-3, C-4, C-5, and UN, and when we targeted L relatives, the competing relationships were L-1, L-2, L-3, and UN.

Although there are a number of publications concerning the strength of evidence, judgment criteria have not been standardized. In this study, we adopted Hummel’s predicates [14] as the criteria for judging kinship determination, and also investigated the range of *ICS* values in each Hummel’s predicate using *ICS* probability distributions.

## Supporting Information

S1 Fig Boxplots of *ics*(*Th*) changing *Th* from 0 to 63 cM. Plots for (A) collateral relatives and (B) lineal relatives.
(DOCX)

S1 Table Means of the AUC values changing *Th*. (DOCX)

S2 Table Estimated values of μ and σ for each relationship in the log-normal distribution. (DOCX)

S3 Table Posterior probabilities from actual sample pairs. (XLSX)
