# Supplementary figures and images for: Pairwise Kinship Analysis by the Index of Chromosome Sharing Using High-Density Single Nucleotide Polymorphisms
Source: PLoS One. 2016 Jul 29;11(7):e0160287. doi: 10.1371/journal.pone.0160287 (PMC4966930; doi:10.1371/journal.pone.0160287)

S1 Fig

1. Collateral relatives


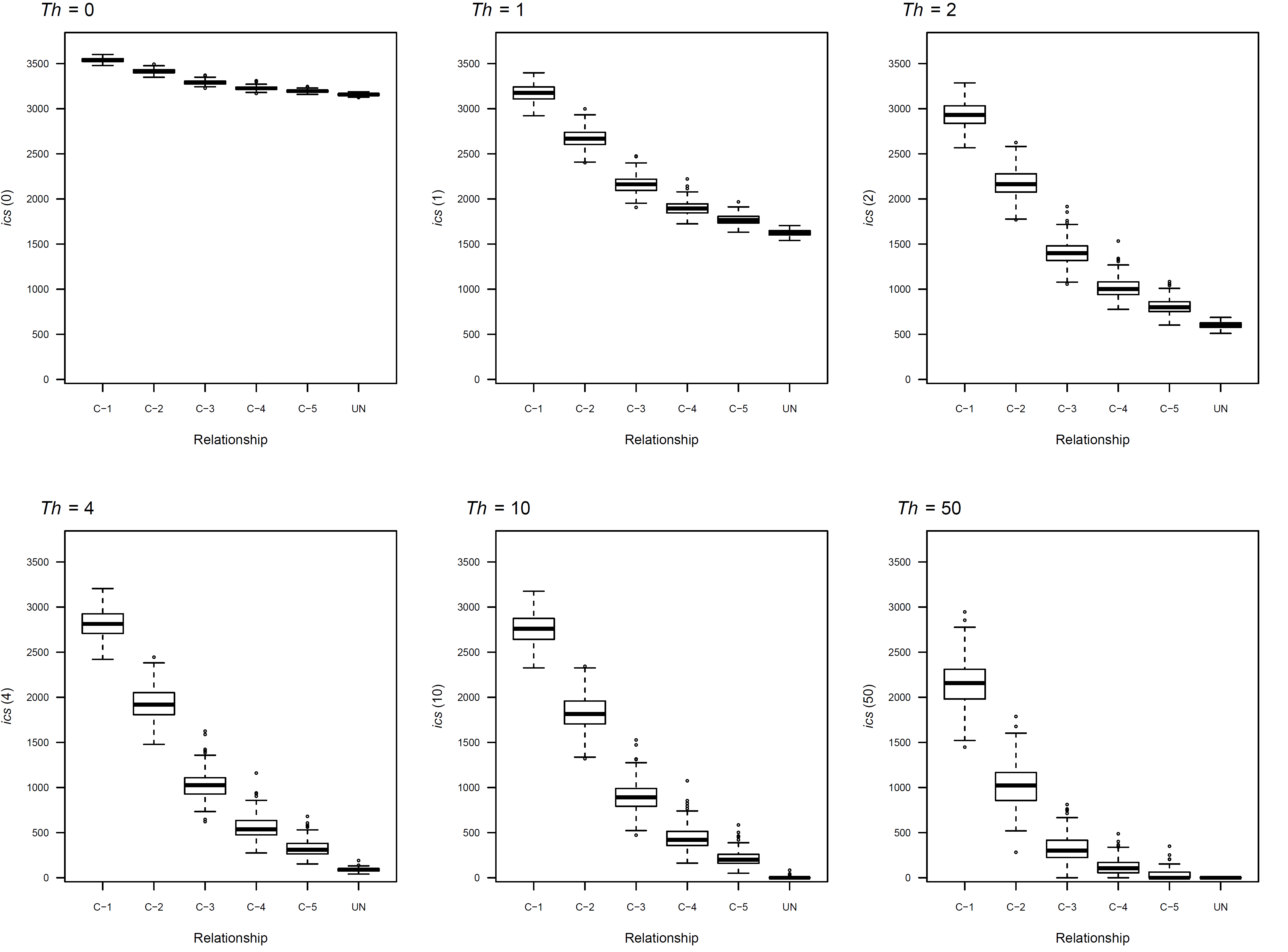


1. Lineal relatives


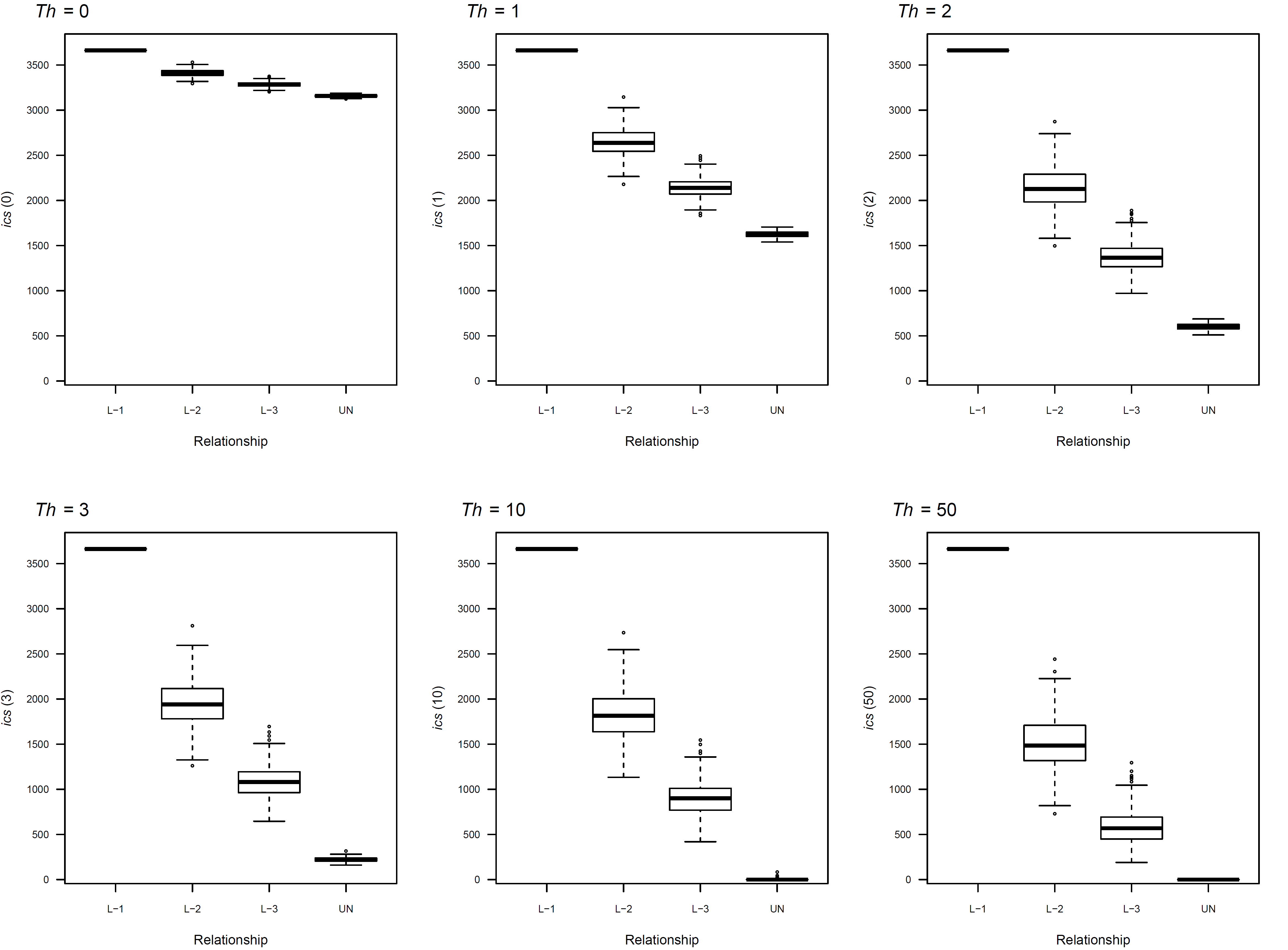

Supplement: S1 Fig — Plots for (A) collateral relatives and (B) lineal relatives. (DOCX) [file pone.0160287.s001.docx]
