# Supplementary material for: Pairwise Kinship Analysis by the Index of Chromosome Sharing Using High-Density Single Nucleotide Polymorphisms
Source: PLoS One. 2016 Jul 29;11(7):e0160287. doi: 10.1371/journal.pone.0160287 (PMC4966930; doi:10.1371/journal.pone.0160287)
Supplement: S1 Table — (DOCX) [file pone.0160287.s002.docx]

S1 Table

| *Th* (cM) | Mean of AUCs | |
| --- | --- | --- |
|  | Collateral relatives | Lineal relatives |
| 0 | 0.97572 | 0.99856 |
| 1 | 0.97851 | 0.99862 |
| 2 | 0.98265 | 0.99885 |
| 3 | 0.98605 | 0.99890 |
| 4 | 0.98646 | 0.99877 |
| 5 | 0.98634 | 0.99887 |
| 6 | 0.98605 | 0.99882 |
| 7 | 0.98556 | 0.99880 |
| 8 | 0.98531 | 0.99881 |
| 9 | 0.98488 | 0.99884 |
| 10 | 0.98442 | 0.99883 |
| 20 | 0.97632 | 0.99876 |
| 50 | 0.87421 | 0.99819 |

AUC, area under the curve; *Th*, threshold.
