# Supplementary material for: Pairwise Kinship Analysis by the Index of Chromosome Sharing Using High-Density Single Nucleotide Polymorphisms
Source: PLoS One. 2016 Jul 29;11(7):e0160287. doi: 10.1371/journal.pone.0160287 (PMC4966930; doi:10.1371/journal.pone.0160287)
Supplement: S2 Table — (DOCX) [file pone.0160287.s003.docx]

S2 Table

Collateral relatives

| Relationship | μ | σ |
| --- | --- | --- |
| C-1 | 7.94 | 0.0565 |
| C-2 | 7.56 | 0.0927 |
| C-3 | 6.93 | 0.151 |
| C-4 | 6.30 | 0.228 |
| C-5 | 5.74 | 0.272 |
| UN | 4.47 | 0.217 |

Lineal relatives

| Relationship | μ | σ |
| --- | --- | --- |
| L-2 | 7.57 | 0.129 |
| L-3 | 6.98 | 0.169 |
| UN | 5.39 | 0.112 |

UN, unrelated.
